# Relationship among atrial fibrillation, the CHA_2_DS_2_-VASc score and ischaemic stroke in patients with coronary artery disease: a propensity score matching study in Hebei, China

**DOI:** 10.1186/s12872-021-02276-z

**Published:** 2021-10-02

**Authors:** Boqun Shi, Demin Liu, Qian Wang, Xue Geng, Qian Hou, Guoqiang Gu, Ruiqin Xie, Wei Cui

**Affiliations:** 1 First Division, Department of Cardiology, The Second Hospital of Hebei Medical University and Institute of Cardiocerebrovascular Disease of Hebei Province, No. 215, Heping West Road, Shijiazhuang, 050000 Hebei China; 2 Department of Cardiology, Coronary Heart Disease Center, Fuwai Hospital, State Key Laboratory of Cardiovascular Disease, National Center for Cardiovascular Diseases, Chinese Academy of Medical Sciences and Peking Union Medical College, Beijing, 100037 China

**Keywords:** Atrial fibrillation, CHA_2_DS_2_-VASc score, Coronary artery disease, Ischaemic stroke

## Abstract

**Background:**

Recent evidence has shown that the pathogenesis of ischaemic stroke associated with atrial fibrillation (AF) is complex and involves other factors in addition to arrhythmias. The purpose of this study was to investigate the relationship among AF, CHA_2_DS_2_-VASc score and ischaemic stroke in patients with coronary artery disease (CAD) in Hebei, China.

**Methods:**

A total of 2,335 patients with CAD from September 2016 to May 2019 at the Second Hospital of Hebei Medical University were included (mean age 62.73 ± 10.35 years, range 26–92 years; 41.58% female). This was a cross-sectional study, and participants were divided into non-stroke (n = 1997) and ischaemic stroke groups (n = 338). Propensity score matching (PSM) was performed to match ischaemic stroke patients with non-stroke patients in a 1:4 ratio. The relationship among AF, the CHA_2_DS_2_-VASc score and ischaemic stroke was evaluated using univariable generalized linear models for different sex, age, body mass index (BMI), CAD and CHA_2_DS_2_-VASc score subgroups. Univariable and multivariable generalized linear models were used to evaluate the relationship between AF and ischaemic stroke in the different models.

**Results:**

Compared with that in the non-stroke group, the prevalence of AF (8.81% vs. 14.20%, *P* = 0.002) in the ischaemic stroke group was higher. The proportion of patients with ischaemic stroke was significantly different between the AF group and the non-AF group (28.74% vs. 19.04%, *P* = 0.003). An increasing CHA_2_DS_2_-VASc score was associated with a gradual increase in the prevalence of AF (*P* for trend < 0.001). Subgroup analysis showed that the trend towards increased stroke risk in the AF group was consistent across the various subgroups. The multivariable analysis demonstrated that AF was not associated with ischaemic stroke compared with the absence of AF (OR = 1.55, 95% CI 0.94–2.56, *P* = 0.087).

**Conclusion:**

In our cross-sectional study, after adjustment for confounding factors, there was no association between AF and ischaemic stroke. The increased risk of ischaemic stroke associated with AF was attenuated by atherosclerotic factors. Our study supports the current view that enhanced control of modifiable cardiovascular risk factors in patients with AF is essential.

## Background

Atrial fibrillation (AF) is a common arrhythmia in clinical practice [1–3]. According to the data of the Global Burden of Disease Study 2017, the age-standardized AF prevalence, incidence, and mortality rates were 0.48%, 0.04% and 0.004%, respectively [4]. In a national cross-sectional study including 31,230 participants from all 31 provinces in China, the prevalence of AF in Chinese adults over 35 years of age was 0.71% [5]. A study in 81,103 male coal miners in Hebei Province found that the prevalence of AF was 0.49%. The mean CHADS_2_ score in AF patients was 0.73. Participants with AF had a significantly higher prevalence of ischaemic stroke than those without AF (7.82% vs. 2.50%, *P* = 0.001) [6]. Although evidence in recent decades has indicated a strong association between AF and ischaemic stroke [7, 8], a gap remains in our understanding of the mechanism underlying this association. The current view is that AF causes blood clotting and thrombus detachment, resulting in cerebral embolism [8]. This mechanism has long been considered the leading cause of ischaemic stroke [9]. However, recent studies have shown that the pathogenesis of ischaemic stroke associated with AF is more complex than previously thought and involves other factors in addition to arrhythmias [10]. Coronary artery disease (CAD) and ischaemic stroke have similar risk factors [11], such as hypertension and advanced age. The purpose of this study was to explore the relationship among AF, CHA_2_DS_2_-VASc score and ischaemic stroke in patients with coronary heart disease and to further understand the role of AF in ischaemic stroke in patients with CAD. The results will have far-reaching significance for future treatment strategies.

## Materials and methods

### Study population

This study was a cross-sectional study conducted according to the Strengthening the Reporting of Observational studies in Epidemiology guidelines. A total of 5583 patients were admitted to the first department of the Cardiovascular Department of the Second Hospital of Hebei Medical University for cardiovascular reasons from September 2016 to May 2019.

**Fig. 1:**
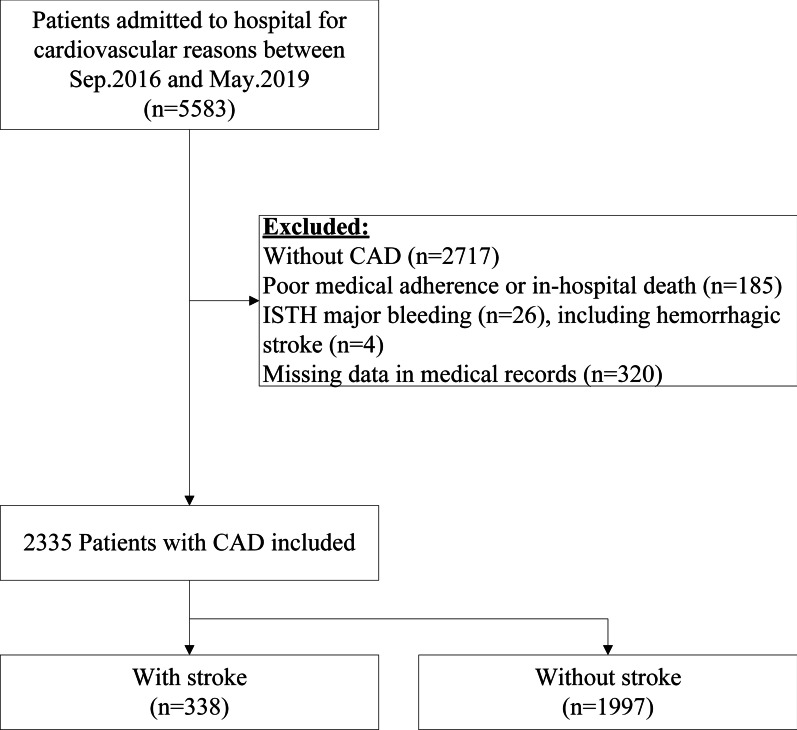
Patient selection flowchart

Patient demographic information, medical histories and laboratory assessments were collected and recorded. As shown in Fig. 1, the exclusion criteria were no CAD (n = 2717), poor medical adherence or in-hospital death (n = 185), major bleeding according to the International Society on Thrombosis and Haemostasis (ISTH) (n = 26), or missing medical record data (n = 320). Finally, 2335 patients (mean age 62.73 ± 10.35 years, range 26–92 years; 41.58% female) were included in this analysis.

The study population was divided into 20 subgroups according to sex, age, body mass index (BMI), type of CAD, and CHA_2_DS_2_-VASc_2_ score ≥ 2 (male) or ≥ 3 (female).

CAD was defined as significant narrowing (≥ 50%) of the coronary lumen detected either invasively or noninvasively; it was further defined as one or more of the following types: stable coronary artery disease (SCAD), unstable angina (UA), non-ST-elevation myocardial infarction (NSTEMI), ST-elevation myocardial infarction (STEMI), previous myocardial infarction (previous MI), and ischaemic cardiomyopathy. Specific diagnoses were performed on the basis of standard reference guidelines.

In this study, ischaemic stroke was defined as a history of ischaemic stroke or new-onset ischaemic stroke during hospitalization.

AF was classified into paroxysmal, persistent, long-standing persistent and permanent AF, which were determined according to the 2014 American College of Cardiology (ACC)/American Heart Association (AHA)/Heart Rhythm Society (HRS) Task Force on Practice Guidelines and the 2016 European Society of Cardiology (ESC) guidelines [12, 13]. In this study, persistent, long-standing persistent, and permanent AF were grouped and collectively referred to as non-paroxysmal AF.

The CHA_2_DS_2_-VASc score is a clinical prediction tool commonly used to identify the risk of ischaemic stroke in patients with nonvalvular AF; it is calculated by assigning 2 points for ischaemic stroke or age ≥ 75 years and 1 point for each additional component, including heart failure, hypertension, vascular disease, age 65–74 years, female sex and diabetes. The CHA_2_DS_2_-VASc score was calculated for each patient at discharge.

We defined diabetes mellitus (DM) as a casual plasma glucose concentration ≥ 200 mg/dL, or fasting plasma glucose concentration ≥ 126 mg/dL, or 2-h plasma glucose concentration ≥ 200 mg/dL according to a 75 g oral glucose tolerance test or drug or insulin use for DM. Hypertension was defined as systolic blood pressure ≥ 140 mmHg, or diastolic blood pressure ≥ 90 mmHg or antihypertensive medication use. Dyslipidaemia was defined as low-density lipoprotein cholesterol (LDL-C) ≥ 140 mg/dL, or high-density lipoprotein cholesterol (HDL-C) < 40 mg/dL, or triglycerides > 150 mg/dL. Heart failure included past or current signs and symptoms of heart failure with both low (< 40%) and preserved ejection fractions (EFs) (≥ 40%) and/or other clinical evidence of cardiac dysfunction. Chronic kidney disease (CKD) was defined as an estimated glomerular filtration rate < 60 mL/min per 1.73 m^2^. Left atrial diameter (LA) and EF were measured by transthoracic echocardiography or transoesophageal echocardiography. Pulse wave velocity (PWV) was defined as the velocity of pulse wave propagation between two fixed points in the arterial system. The ankle-brachial index (ABI) was calculated as the ratio between the ankle artery pressure and the brachial artery pressure. Both PWV and ABI were measured by a specially trained nurse using PWV/ABI instruments (form PWV/ABI, BP-203RPE; Omron-Colin, Japan).

### Statistical analysis

The investigators performed the data analysis using Empower (R) (www.empowerstats.com, X&Y solutions, Inc. Boston, Massachusetts, USA) and R 3.6.3 (http://www.R-project.org). The Shapiro–Wilk test was used to examine whether continuous variables were normally distributed. Continuous variables in this study that did not conform to a normal distribution are presented as medians and interquartile ranges (IQRs) and were compared using the Mann–Whitney test. Categorical variables are presented as counts and composition ratios and were compared using the chi-square test or Fisher’s exact test as appropriate.

Given the inherent differences between patients with and without ischaemic stroke, we calculated a propensity score for the following variables: sex, age, and BMI. The propensity score was estimated by a logistic regression model. The matching process was based on the nearest-neighbour matching method without replacement within a calliper of 0.01; participants were 1:4 matched, which yielded a stroke group and a matched non-stroke group.

To control for potential covariates, we used multivariable generalized linear regression models. The multivariable analysis was adjusted for age, hypertension, aortic atherosclerosis, AF, ABI, CKD, PWV, LA, STEMI, heart failure, previous MI, ischaemic cardiomyopathy, smoking, alcohol consumption, and DM. These variables were selected on the basis of statistical significance (*P* < 0.1) in the univariable analysis or previous reports or because they are empirically associated with stroke. Variables with multicollinearity were excluded. The Cochran–Mantel–Haenszel test was used in the analysis to calculate the P value for trend. The likelihood ratio test was performed after adjustment for subgroup factors. A two-tailed *P* value < 0.05 was considered statistically significant in this study.

## Results

### Patient characteristics

A total of 2335 patients with CAD, including 1997 patients without ischaemic stroke (85.52%) and 338 patients with ischaemic stroke (14.48%), were enrolled in this study. Three (0.89%) of the ischaemic stroke patients had acute stroke, while the remaining 335 (99.11%) had prior stroke. The matched cross-sectional analysis included 1,685 patients (1348 patients without ischaemic stroke and 337 patients with ischaemic stroke). The patients’ baseline characteristics before and after PSM are shown in Table 1.

**Table 1:** Comparison of baseline characteristics

	Before PSM			After PSM		
	Without stroke (n = 1997)	With stroke (n = 338)	*P* value	Without stroke (n = 1348)	With stroke (n = 337)	*P* value
Sex			0.771			0.980
Female	828 (41.46%)	143 (42.31%)		573 (42.51%)	143 (42.43%)	
Male	1169 (58.54%)	195 (57.69%)		775 (57.49%)	194 (57.57%)	
Age (years)	63.00 (55.00–69.00)	65.00 (60.00–72.00)	< 0.001*	64.00 (58.00–70.00)	65.00 (60.00–72.00)	< 0.001*
BMI (kg/m^2^)	25.39 (23.44–27.68)	25.50 (23.23–27.68)	0.929	25.39 (23.29–27.68)	25.50 (23.23–27.68)	0.941
CAD type						
SCAD	128 (6.41%)	36 (10.65%)	0.005*	86 (6.38%)	36 (10.68%)	0.006*
UA	1471 (73.66%)	229 (67.75%)	0.024*	989 (73.37%)	229 (67.95%)	0.047*
NSTEMI	286 (14.32%)	41 (12.13%)	0.283	201 (14.91%)	41 (12.17%)	0.199
STEMI	79 (3.96%)	23 (6.80%)	0.018*	51 (3.78%)	22 (6.53%)	0.027*
Previous MI	205 (10.27%)	49 (14.50%)	0.021*	146 (10.83%)	49 (14.54%)	0.057
Ischaemic cardiomyopathy	34 (1.70%)	11 (3.25%)	0.082	23 (1.71%)	11 (3.26%)	0.069
Heart failure	312 (15.62%)	72 (21.30%)	0.009*	228 (16.91%)	72 (21.36%)	0.056
Hypertension	1307 (65.45%)	262 (77.51%)	< 0.001*	899 (66.69%)	262 (77.74%)	< 0.001*
Dyslipidaemia	605 (30.30%)	95 (28.11%)	0.417	414 (30.71%)	94 (27.89%)	0.313
Total cholesterol (mmol/L)	3.99 (3.40–4.67)	3.94 (3.32–4.76)	0.544	4.00 (3.37–4.68)	3.94 (3.32–4.76)	0.478
LDL-C (mmol/L)	2.51 (1.92–3.11)	2.45 (1.91–3.15)	0.615	2.51 (1.90–3.11)	2.45 (1.91–3.15)	0.567
Valvular heart disease	43 (2.15%)	8 (2.37%)	0.840	32 (2.37%)	8 (2.37%)	1
AF	176 (8.81%)	48 (14.20%)	0.002*	119 (8.83%)	48 (14.24%)	0.003*
AF type			< 0.001*			0.003*
No	1821 (91.19%)	290 (85.80%)		1229 (91.17%)	289 (85.76%)	
paroxysmal AF	106 (5.31%)	22 (6.51%)		69 (5.12%)	22 (6.53%)	
Non-paroxysmal AF	70 (3.51%)	26 (7.69%)		50 (3.71%)	26 (7.72%)	
DM	551 (27.59%)	102 (30.18%)	0.327	378 (28.04%)	102 (30.27%)	0.418
COPD	40 (2.00%)	7 (2.07%)	0.836	30 (2.23%)	7 (2.08%)	0.868
CKD	39 (1.95%)	13 (3.85%)	0.043*	23 (1.71%)	13 (3.86%)	0.015*
Creatine (μmol/L)	69.00 (59.00–80.95)	72.00 (61.30–86.00)	0.001*	70.00 (59.00–82.00)	72.00 (61.30–86.00)	0.011*
Fatty liver	39 (1.95%)	5 (1.48%)	0.670	30 (2.23%)	5 (1.48%)	0.393
Smoking	409 (20.48%)	57 (16.86%)	0.124	261 (19.36%)	56 (16.62%)	0.249
Alcohol consumption	347 (17.38%)	47 (13.91%)	0.115	224 (16.62%)	46 (13.65%)	0.184
Aortic atherosclerosis	529 (26.81%)	146 (43.84%)	< 0.001*	386 (28.94%)	146 (43.98%)	< 0.001*
LA (mm)	34.00 (32.00–37.00)	35.00 (32.00–37.00)	0.073	34.00 (32.00–37.00)	35.00 (32.00–37.00)	0.024*
EF (%)	61.74 (60.61–62.89)	61.70 (60.64–62.77)	0.574	61.74 (60.61–62.86)	61.70 (60.64–62.77)	0.73
PWV (cm/s)	1556.50 (1367.50–1798.50)	1623.00 (1418.00–1906.50)	< 0.001*	1575.25 (1388.00–1814.38)	1625.00 (1418.00–1908.62)	0.022*
ABI	1.09 (1.02–1.156)	1.08 (0.97–1.16)	0.057	1.08 (1.01–1.16)	1.08 (0.97–1.16)	0.005*
CHA_2_DS_2_-VASc score	3.00 (2.00–4.00)	4.00 (3.00–5.00)	< 0.001*	3.00 (2.00–4.00)	4.00 (3.00–5.00)	< 0.001*
Propensity score	0.16 ± 0.04	0.14 ± 0.04	< 0.001*	0.16 ± 0.04	0.15 ± 0.04	< 0.001*

*Indicates *p* < 0.05

*BMI* body mass index, *CAD* coronary artery disease, *SCAD* stable coronary artery disease, *UA* unstable angina, *NSTEMI* non-ST-elevation myocardial infarction, *STEMI* ST-elevation myocardial infarction, *previous MI* previous myocardial infarction, *AF* atrial fibrillation, *DM* diabetes mellitus, *COPD* chronic obstructive pulmonary disease, *CKD* chronic kidney disease, *LDL-C* low-density lipoprotein cholesterol, *LA* left atrial diameter, EF ejection fraction, *PWV* pulse wave velocity, *ABI* ankle-brachial index

Before PSM, compared with the non-stroke group, the ischaemic stroke group was older (63.00 vs. 65.00, *P* < 0.001), and the proportions of SCAD (6.41% vs. 10.65%, *P* = 0.005), STEMI (3.96% vs. 6.80%, *P* = 0.018), and previous MI (10.27% vs. 14.50%, *P* = 0.021) were higher. The proportion of UA (73.66% vs. 67.75%, *P* = 0.024) was lower, and the proportions of heart failure (15.62% vs. 21.30%, *P* = 0.009), hypertension (65.45% vs. 77.51%, *P* < 0.001), AF (8.81% vs. 14.20%, *P* = 0.002), CKD (1.95% vs. 3.85%, *P* = 0.043), and aortic atherosclerosis (26.81% vs. 84%, *P* < 0.001) were higher. The creatinine level, PWV and CHA_2_DS_2_-VASc score were also higher. These differences were statistically significant.

After PSM, compared with those in the non-stroke group, the proportions of STEMI, UA, hypertension, AF, and aortic atherosclerosis were higher in the ischaemic stroke group. The serum creatinine level and CHA_2_DS_2_-VASc score were also higher. These differences were statistically significant.

A comparison of the baseline data of patients with and without AF after PSM is shown in Table 2. Compared with the non-AF group, the AF group was older (63.50 vs. 69.00, *P* < 0.001); had higher prevalence rates of SCAD (4.61% vs. 31.14%, *P* < 0.001), previous MI (10.94% vs. 17.37%, *P* = 0.014), ischaemic cardiomyopathy (1.58% vs. 5.99%, *P* < 0.001), and UA (74.57% vs. 51.50%); had lower prevalence rates of STEMI (4.68% vs. 1.20%, *P* = 0.036), heart failure (13.90% vs. 53.29%, *P* < 0.001), valvular heart disease (1.91% vs. 6.59%, *P* < 0.001), CKD (1.84% vs. 4.79%, *P* = 0.012), smoking (19.50% vs. 12.57%, *P* = 0.03), and aortic atherosclerosis (30.53% vs. 44.58%); had a higher serum creatinine level; had a larger LA; and had a higher PWV and CHA_2_DS_2_-VASc score. Furthermore, the ABI and EF were lower. These differences were statistically significant. The proportion of patients with ischaemic stroke was significantly different between the two groups (19.04% vs. 28.74%, *P* = 0.003).

**Table 2:** Comparison of baseline characteristics after PSM with and without AF

	Without (n = 1518)	With AF (n = 167)	*P* value
Sex			0.505
Female	641 (42.23%)	75 (44.91%)	
Male	877 (57.77%)	92 (55.09%)	
Age (years)	63.50 (58.00–70.00)	69.00 (63.00–77.00)	< 0.001*
BMI (kg/m^2^)	25.39 (23.23–27.68)	25.69 (23.44–27.68)	0.872
CAD type			
SCAD	70 (4.61%)	52 (31.14%)	< 0.001*
UA	1132 (74.57%)	86 (51.50%)	< 0.001*
NSTEMI	225 (14.82%)	17 (10.18%)	0.104
STEMI	71 (4.68%)	2 (1.20%)	0.036*
Previous MI	166 (10.94%)	29 (17.37%)	0.014*
Ischaemic cardiomyopathy	24 (1.58%)	10 (5.99%)	< 0.001*
Heart failure	211 (13.90%)	89 (53.29%)	< 0.001*
Hypertension	1041 (68.58%)	120 (71.86%)	0.385
Dyslipidaemia	456 (30.04%)	52 (31.14%)	0.769
Total cholesterol (mmol/L)	4.00 (3.39–4.70)	3.83 (3.28–4.70)	0.229
LDL-C (mmol/L)	2.52 (1.91–3.12)	2.32 (1.88–3.09)	0.301
Valvular heart disease	29 (1.91%)	11 (6.59%)	< 0.001*
DM	430 (28.33%)	50 (29.94%)	0.661
COPD	35 (2.31%)	2 (1.20%)	0.354
CKD	28 (1.84%)	8 (4.79%)	0.012*
Creatine (μmol/L)	69.00 (59.00–81.00)	79.85 (68.00–94.75)	< 0.001*
Fatty liver	33 (2.17%)	2 (1.20%)	0.401
Smoking	296 (19.50%)	21 (12.57%)	0.03*
Alcohol consumption	249 (16.40%)	21 (12.57%)	0.201
Aortic atherosclerosis	458 (30.53%)	74 (44.58%)	< 0.001*
LA (mm)	34.00 (32.00–36.00)	38.00 (35.00–42.50)	< 0.001*
EF (%)	61.74 (60.64–62.89)	60.78 (55.48–62.05)	0.001*
PWV (cm/s)	1565.00 (1387.75–1810.75)	1734.75 (1505.88–2149.62)	< 0.001*
ABI	1.08 (1.00–1.16)	1.06 (0.94–1.14)	0.004*
CHA_2_DS_2_-VASc score	3.00 (2.00–4.00)	4.00 (3.00–6.00)	< 0.001*
Stroke	289 (19.04%)	48 (28.74%)	0.003*

*Indicates *p* < 0.05

*BMI* body mass index, *CAD* coronary artery disease, *SCAD* stable coronary artery disease, *UA* unstable angina, *NSTEMI* non-ST-elevation myocardial infarction, *STEMI* ST-elevation myocardial infarction, previous *MI* previous myocardial infarction, *AF* atrial fibrillation, *DM* diabetes mellitus, *COPD* chronic obstructive pulmonary diseases, *CKD* chronic kidney disease, *LDL-C *low-density lipoprotein cholesterol, *LA* left atrial diameter, *EF* ejection fraction, *PWV* pulse wave velocity, *ABI* ankle-brachial index

### Subgroup analysis

**Fig. 2:**
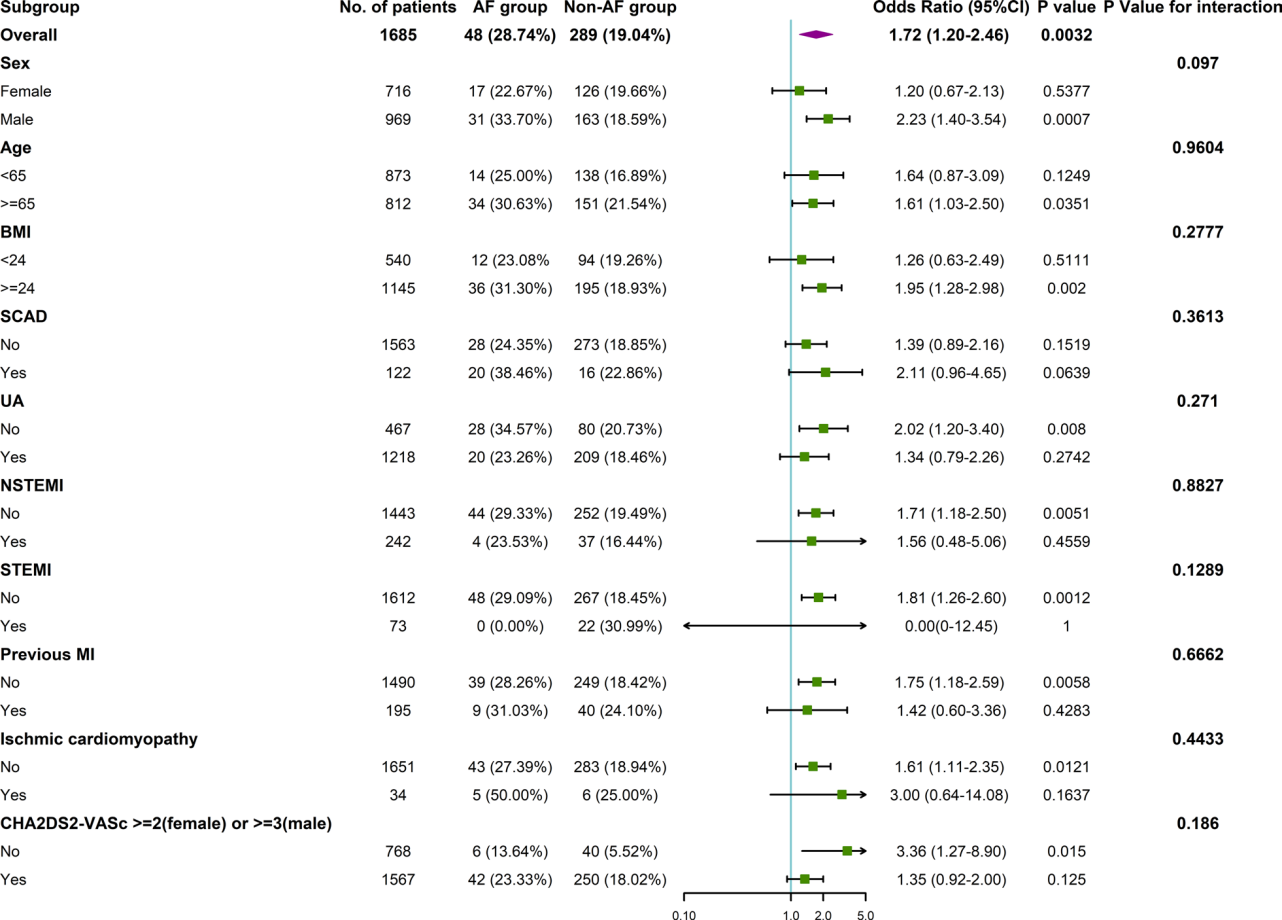
Association between AF and ischaemic stroke in prespecified and exploratory subgroups after PSM. *P* for interaction is based on the likelihood ratio test

Subgroup analysis according to baseline characteristics between the AF group and the non-AF group was performed (Fig. 2). A trend towards an increased risk of stroke in the AF group was observed in the following subgroups: male, age ≥ 65 years, BMI ≥ 24 kg/m^2^, non-UA, non-NSTEMI, non-STEMI, non-previous MI, non-ischaemic cardiomyopathy and CHA_2_DS_2_-VASc < 2(male) or < 3(female) subgroups. Interestingly, AF seemed to have no association with stroke in the CHA_2_DS_2_-VASc ≥ 2 (female) or ≥ 3 (male) subgroup (OR = 1.35, 95% CI 0.92–2.00, *P* = 0.125, *P* for interaction = 0.186). However, the subgroup analyses did not indicate any significant interactions between ischaemic stroke and the stratification variables.

### Association between AF and ischaemic stroke

The univariable analysis of the relationship between AF and ischaemic stroke after PSM (Table 3) showed that AF was associated with ischaemic stroke (OR = 1.71, 95% CI 1.20–2.46, *P* = 0.003). However, the multivariable analysis showed that AF was not associated with ischaemic stroke compared with no AF (OR = 1.55, 95% CI 0.94–2.56, *P* = 0.087). The multivariable analysis was adjusted for age, hypertension, aortic atherosclerosis, AF, ABI, CKD, PWV, LA, STEMI, heart failure, previous MI, ischaemic cardiomyopathy, smoking, alcohol consumption, and DM.

**Table 3:** Univariable and multivariable analysis of the association between AF and ischaemic stroke after PSM

Variable	Univariable OR (95% CI) *P* value	Multivariable OR (95% CI) *P* value
Without AF	1.00	1.00
With AF	1.71 (1.20–2.46) 0.003*	1.55 (0.94–2.56) 0.087

*Indicates *p* < 0.05

The multivariable analysis was adjusted for age, hypertension, aortic atherosclerosis, AF, ABI, CKD, PWV, LA, STEMI, heart failure, previous MI, ischaemic cardiomyopathy, smoking, alcohol consumption, and DM

### Association between AF burden and ischaemic stroke

**Fig. 3:**
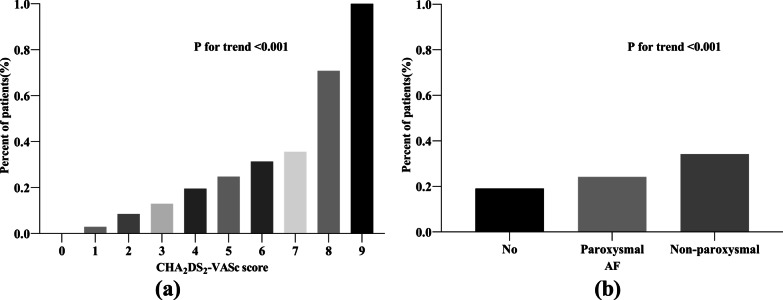
**a** Association between the CHA_2_DS_2_-VASc score and AF. **b** Association between AF type and ischaemic stroke

The univariable analysis of the relationship between the AF burden and ischaemic stroke after PSM (Table 4) showed that compared with paroxysmal AF, non-paroxysmal AF did not increase the prevalence of ischaemic stroke (OR = 1.63, 95% CI 0.83–3.20, *P* = 0.155). The multivariable analysis produced similar results (OR = 1.02, 95% CI 0.32–3.24, *P* = 0.973). However, as shown in Fig. 3b, an increasing AF burden was associated with a continuous increase in the prevalence of ischaemic stroke (*P* for trend < 0.001).

**Table 4:** Univariable and multivariable analysis of the association between AF burden and ischaemic stroke after PSM

Variable	Univariable OR (95% CI) P value	Multivariable OR (95% CI) P value
Paroxysmal AF	1.00	1.00
Non-paroxysmal AF	1.63 (0.83–3.20) 0.155	1.02 (0.32–3.24) 0.973

*Indicates *p* < 0.05

The multivariable analysis was adjusted for age, hypertension, aortic atherosclerosis, AF, ABI, CKD, PWV, LA, STEMI, heart failure, previous MI, ischaemic cardiomyopathy, smoking, alcohol consumption, and DM

### Relationship among CHA_2_DS_2_-VASc score and ischaemic stroke

An increasing CHA_2_DS_2_-VASc score was associated with a gradual increase in the prevalence of AF (*P* for trend < 0.001, Fig. 3a).

## Discussion

### AF and ischaemic stroke have common risk factors

The results of this study suggest that age, heart failure, hypertension, CKD, creatinine level, aortic atherosclerosis, PWV, and the CHA_2_DS_2_-VASc score are associated with ischaemic stroke and AF.

Previous studies have shown that age, male sex, hypertension, DM, valvular heart disease, heart failure, CAD, CKD, inflammatory disease, obstructive sleep apnoea syndrome and smoking are common risk factors for AF and ischaemic stroke [12, 14, 15]. Potential risk factors, such as obesity and metabolic syndrome, contribute to the development of AF and atrial cardiomyopathy through a variety of mechanisms [16]. The Framingham Heart Study [17] reported that the lifetime risk of AF in those with an optimal risk profile was approximately 20%, which was lower than that in those with a high-risk profile (38.4%). The UK Biobank study [16] confirmed that there were significant differences in AF risk among the ideal lifestyle group, the general lifestyle group, and the poor lifestyle group, regardless of genetic risk. As more common risk factors were considered, the association between AF and ischaemic stroke was reduced, suggesting confounding.

### Possible mechanism of ischaemic stroke in patients with CAD complicated with AF

The univariable analysis showed that in patients with CAD, AF was associated with ischaemic stroke; however, the association between AF and ischaemic stroke disappeared in the multivariable analysis, which included adjustment for other risk factors. In the subgroup analysis, AF appeared to have no association in the CHA_2_DS_2_-VASc ≥ 2 (female) or ≥ 3 (male) subgroup. It is speculated that the risk of ischaemic stroke does not increase after the CHA_2_DS_2_-VASc score reaches a certain threshold in patients with CAD. Furthermore, comparing a group with CAD plus AF to a group with AF without CAD would be important for determining whether atherosclerotic factors play important roles in ischaemic stroke. A report of the Western Denmark Heart Registry [18] demonstrated that CAD was an independent risk factor for a composite thromboembolic outcome in addition to the risk factors already included in the CHA_2_DS_2_-VASc score. As a result, CAD diagnosed by coronary angiography has been added as a vascular disease criterion in the CHA_2_DS_2_-VASc score [19]. Our outcome is contrary to that of a meta-analysis by Santarpia et al., which showed that oral anticoagulant use after successful radiofrequency cardioversion of AF did not reduce the prevalence of embolic events [20]. A possible explanation might be as follows: both AF and atherosclerosis are independent risk factors for stroke, and the hazard ratios of these two risk factors for stroke vary among individuals. Antithrombotic therapy after successful radiofrequency ablation should be determined based on the patient's baseline risk of thromboembolism and bleeding. Large-scale randomized controlled trials are needed to determine the best anticoagulant therapy regimen for patients with AF following successful radiofrequency ablation to achieve the greatest clinical net benefit.

### Association between AF burden and ischaemic stroke

In this study, both the univariable and multivariable analyses showed that non-paroxysmal AF does not increase the prevalence of ischaemic stroke compared with paroxysmal AF. However, the trend results showed that with increasing AF burden, the prevalence of ischaemic stroke increased continuously. These inconsistent findings suggest that compared with patients with paroxysmal AF, patients with non-paroxysmal AF do not appear to have an increased risk of ischaemic stroke. Our study did not provide sufficient evidence that the AF burden is positively associated with ischaemic stroke.

Although many studies have found a dose–response relationship between AF load and ischaemic stroke [21–23], the results are inconsistent. A recent KP-Rhythm study identified a higher risk of stroke in the high-load group (> 11%, approximately 2.5 h/24 h) [23]. A study including 384 patients with cardiovascular implantable electronic device-detected AF not receiving oral anticoagulation suggested that the duration of AF was not associated with the risk of stroke, whereas the CHA_2_DS_2_-VASc score was [24]. There is no standardized AF burden threshold to predict a significantly increased risk of thrombosis. The current guidelines [12, 13] generally consider that if the duration of AF is less than 48 h, there is no need to perform transoesophageal echocardiography. Regardless of the CHA_2_DS_2_-VASc score and the method used to restore the sinus rhythm, cardioversion can be performed without anticoagulation. However, whether the 48-h threshold applies to all patients with AF is debatable [25]. In addition, a brief episode of subclinical AF doubled the risk of ischaemic stroke in elderly patients [26]. while there was no significant increase in the risk of ischaemic stroke in young, healthy patients with AF [27]. Additionally, atrial remodelling in animal models of AF occurred at least one week after continuous rapid pacing [28]. Therefore, any atrial changes caused by AF are unlikely to explain the association between a short (6-min) AF episode and an increased risk of ischaemic stroke [26]. Overall, these conflicting data are insufficient to establish a clear dose–response relationship between AF burden and the risk of ischaemic stroke.

### Study strength and limitations

The novelty of this study is the finding that among patients with CAD, who tend to have a higher degree of atherosclerosis than patients with only AF, the risk of ischemic stroke associated with AF was determined by atherosclerotic factors.

There are several limitations to our research. First, it was a cross-sectional study, and ischaemic stroke, the CHA_2_DS_2_-VASc score, and AF were measured at the same time. In addition, several variables remained imbalanced after PSM. Therefore, we also performed multivariable analysis to minimize bias.

## Conclusion

In our cross-sectional study, after adjustment for confounding risk factors, the association between AF and ischaemic stroke in patients with CAD disappeared. In the subgroup analysis, AF appeared to have no association with stroke in the CHA_2_DS_2_-VASc ≥ 2 (female) or ≥ 3 (male) subgroup. The increased risk of ischemic stroke associated with AF was determined by atherosclerotic factors. Our study supports the current view that enhanced control of modifiable cardiovascular risk factors in patients with AF is essential.

## Data Availability

The datasets used and/or analysed during the current study are available from the corresponding author upon reasonable request.

## Abbreviations

BMI: body mass index
CAD: coronary artery disease
SCAD: stable coronary artery disease
UA: unstable angina
NSTEMI: non-ST-elevation myocardial infarction
STEMI: ST-elevation myocardial infarction
previous MI: previous myocardial infarction
AF: atrial fibrillation
DM: diabetes mellitus
COPD: chronic obstructive pulmonary disease
CKD: chronic kidney disease
LDL-C: low-density lipoprotein cholesterol
LA: left atrial diameter
EF: ejection fraction
PWV: pulse wave velocity
ABI: ankle-brachial index
